# A Sensor-Type PC Strand with an Embedded FBG Sensor for Monitoring Prestress Forces

**DOI:** 10.3390/s150101060

**Published:** 2015-01-08

**Authors:** Sung Tae Kim, YoungHwan Park, Sung Yong Park, Keunhee Cho, Jeong-Rae Cho

**Affiliations:** Division of Structural Engineering Research/Korea Institute of Construction and Building Technology, 283, Goyangdae-ro, Ilsanseo-gu, Goyang-si, Gyeonggi-do 411-712, Korea; E-Mails: esper009@kict.re.kr (S.T.K.); sypark@kict.re.kr (S.Y.P.); kcho@kict.re.kr (K.C.); chojr@kict.re.kr (J.-R.C.)

**Keywords:** PSC, strand, optical fiber sensor, FBG

## Abstract

Prestressed Concrete Wire and Strand (PC) strands are the most used materials to introduce prestress in a Pre-Stressed Concrete (PSC) structure. However, it is difficult to evaluate the final prestress force of the PC strand after prestressing or its residual prestress force after completion of the structure on site. This impossibility to assess eventual loss of prestress of the PC strand has resulted in a number of serious accidents and even in the collapse of several structures. This situation stresses the necessity to maintain the prestress force residual or after prestressing for the evaluation of the health of the concrete structure throughout its lifespan. Recently, several researchers have studied methods enabling one to verify the prestress force by inserting an optical fiber sensor inside the strand but failed to provide simple techniques for the fabrication of these devices to fulfill measurement performance from the design prestress to failure. Moreover, these methods require the additional installation of electrical resistance strain gages, displacement sensors and load cells on the outer surface of the structure for long-term precise measurement. This paper proposes a method enabling one to evaluate precisely and effectively the prestress force of the PC strand and intends to verify the applicability of the proposed method on actual concrete structures. To that end, an innovative PC strand is developed by embedding a Fiber Bragg Grating (FBG) sensor in the core wire of the PC strand so as to enable short term as well as long term monitoring. The measurement performance of the developed strand is then evaluated experimentally and the reliability of the monitoring data is assessed.

## 1. Introduction

The introduction of prestress in a concrete structure improves its ultimate strength and its deflection and cracking characteristics. PC strands are the most used materials to introduce prestress in a concrete structure. However, it is currently not possible to evaluate the residual prestress force in the PC strand after the prestressing process or after the completion of the structure. This impossibility to identify the loss of prestress caused by insufficient prestressing or the corrosion of the PC strand prevents the deployment of countermeasures and has resulted in serious accidents and even in the collapse of structures [1,2]. This situation stresses the necessity to maintain the prestress force residual or after prestressing for the evaluation of the health of the structure along its whole lifespan.

Recently, Kim *et al.* [3] and Kim *et al.* [4] proposed a method enabling one to assess the prestress force by inserting an optical fiber sensor inside a tube installed in the core wire of the strand. However, these authors failed to provide robust sensing performance data measuring the prestress force from the design prestress to failure. The works of Caro *et al.* [5] and Martí-Vargas *et al.* [6,7] additionally installed electrical resistance strain gages, displacement sensors and load cells at the outer surface of the structure to observe the long-term variation of the prestress force.

This study intends to develop a PC strand with a built-in sensor enabling one to measure the prestress force all along the service life from the start of prestressing to failure of the strand while fulfilling its role as structural member without installing additional sensors outide the structure. The considered strand is a 15.2 mm 7-wire strand with tensile strength of 1860 MPa usually used to apply prestress in PSC structures.

In order to confer measuring capability to the PC strand, we can work with the core wire or the surrounding wires or replace them with another material. This study surveyed previous similar research with focus on the workability, economy and convenience in the installation of the sensor [8–11]. The methods for installing the sensor in the surrounding wire usually utilize a groove or exploit gaps in the wire to embed the sensor and fill it with epoxy. The use of such methods may result in different axial strain measurements due to the differences in length with the core wire since the surrounding wire is wrapped helically around the core wire. Moreover, the tensioning of the strand makes the helical wire spread from its originally bent state, which in turn increases the risk of damage to the filler and, to worsen the situation, the sensor is more vulnerable to direct damage provoked by external impacts.

Accordingly, it appears that embedding the sensor in the core wire should be recommended in view of the orientation of the sensor, its monolithic behavior and the installation convenience and, because the core wire preserves its straightness and is protected from external impact. Besides, according to Kim *et al.* [12]. the method of embedding the optical fiber sensor in a conventional steel core wire by cutting or grooving the wire degrades the performance caused by the loss of cross-sectional area by the steel working and, is costlier due to the high cost required by the additional working process. Therefore, the most appropriate solution would be to fabricate the core wire using another material enabling convenient installation of the sensor. This material should provide performance superior or equivalent to the steel core wire and should allow the fabrication of a core wire with the desired length and a transverse cross section with dimensions identical to the steel core wire. Since previous studies used a tube or glass fiber as core wire, it was impossible to prestress the strand. Form all this it follows that most advantageous material satisfying all these requirements in terms of performance, fabricability and economy is carbon fiber.

Consequently, this study adopts carbon fiber as the material for the core wire for the sensing strand. On the other hand, the small diameter (about 5.2 mm) and the extended length of the core wire of the PC strand necessitate a thin thread-shaped sensor. The emerging FBG sensor, which has started to be applied extensively for the monitoring of recent structures, satisfies these conditions. Since this sensor presents a thin diameter of about 0.15 to 0.25 μm and can be fabricated with the desired length, it is the most suitable for being embedded during the manufacture of the carbon core wire. Furthermore, its outstanding durability is key for long-term monitoring, and its immunity to the effects of surrounding electromagnetic waves ensures the high reliability of its measurements.

Based upon the results of the investigation performed on the fabrication method and materials adopted for the sensing PC strand, the strand is fabricated by replacing the steel core wire by a carbon core wire and the FBG sensor is embedded in the core wire. In addition, the measured data are analyzed to examine their reliability, and the applicability of the new PC strand on real PSC structures is validated.

## 2. Experimental Section

### 2.1. Fabrication of Sensor-Type PC Strand

**Figure 1.**
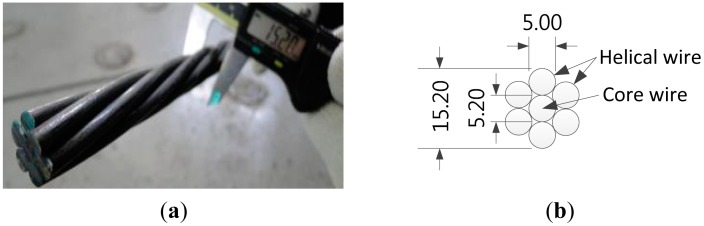
(**a**) Shape and (**b**) dimensions of PC strand.

The PC strand with measuring function must exhibit not only the performance of a sensor but also the structural performance of a steel strand. Therefore, the core wire is made of carbon fiber to achieve performance equivalent or superior to the steel core wire of the conventional PC strand. Since carbon fiber displays outstanding strength, superior to steel, it is the most suitable material to replace steel. Moreover, the adoption of carbon fiber for the manufacture of the core wire enables the embedment of the sensor without need of supplemental processes. Figure 1 presents the shape and dimensions of the strand used in this study. Table 1 lists the specifications of the FBG sensor embedded in the core wire of the strand with measuring function.

The difference in diameter shall be minimized to ease the assemblage of the core wire with the surrounding helical wires. Therefore, the carbon fiber core wire is fabricated by adjusting its diameter to 5.3 mm so as to achieve a diameter of about 5.4–5.5 mm when the external nylon cover fiber is included. Moreover, the volume fraction of the carbon fiber is adjusted to be larger than 65%. The corresponding stiffness of the actually as-fabricated carbon core wire is approximately 163 GPa.

**Figure 2.**
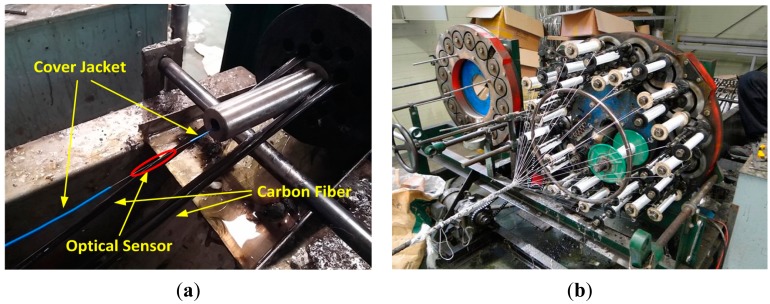
(**a**) Insertion of optical sensor and (**b**) fabrication of carbon core wire.

Besides, in order to realize a sensing strand producing exact prestress force measurements, the optical fiber sensor shall be monolithically composed with the carbon fiber core wire. In addition, the bare cable section of the optical fiber apart from the sensor part is resistant to tension, but extremely weak to shear, which make its handling delicate. Accordingly, the bare section of the optical fiber is protected by a covering jacket and inserted in the center of the core wire. Braidtrusion was chosen as the fabrication method of the core wire because it can apply a constant tensile force in the axial direction while maintaining the straightness of the carbon fiber and, can prevent the galvanic corrosion that can occur through the contact with the outer steel helical wires owing to the external nylon fiber wrapping. Figure 2 shows the fabrication process of the core wire by braidtrusion.

**Figure 3.**
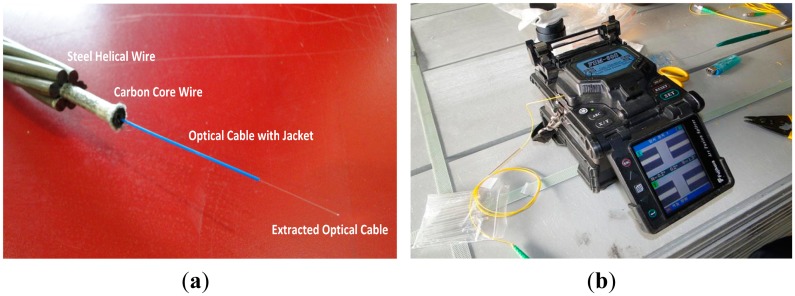
(**a**) Completed sensing strand and (**b**) optic fusion.

In Figure 2, the strand is completed by wrapping the steel helical wires around the carbon core wire. Then, the optical fiber cable protected by the cover jacket is extended outside the core wire for optic fusion work in order to enable the measurement function. Figure 3 illustrates the assembled sensing strand using the completed carbon core wire and the optic fusion work.

### 2.2. Performance Test of the Sensing Strand

**Figure 4.**
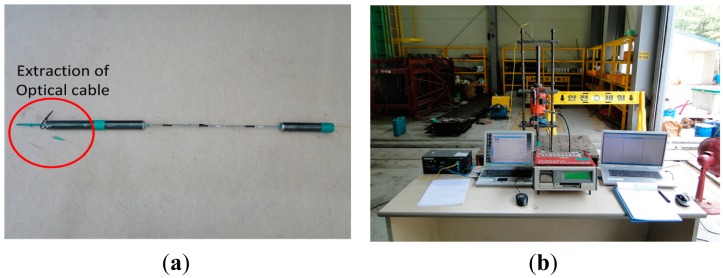
(**a**) Carbon core wire specimen for sensing strand and (**b**) setting of core wire test.

The performance test of the sensing strand is conducted in two stages: stage 1 tests the core wire and stage 2 tests the strand. The specimens are fabricated as shown in Figure 4a to enable direct tensile tests of the core wire. The tensile force is applied in the axial direction using a hydraulic jack as shown in Figure 4b. Electrical resistance strain gauges are bonded on the surface of the core wire for further comparison of the measured data with the optical fiber sensor embedded in the core wire.

For the strand specimens, sets of three electrical resistance strain gauges oriented at 120° with respect to each other in a delta rosette are bonded on each specimen at locations identical to those at which the optical fiber sensor is installed. Comparison is done with the strain of the optical fiber sensor using the average of the strain at three positions. This disposition is arranged to prepare for the eventual occurrence of different strains in the helical wires and the possible differences in the state of the gauges installed on each helical wire. Moreover, the axial strain of an ordinary strand is measured by means of an extensometer installed on the Universal Testing Machine (UTM) for the comparison with the strain measured by the optical fiber sensor of the sensing strand so as to verify the structural performance and the reliability of the data.

**Figure 5.**
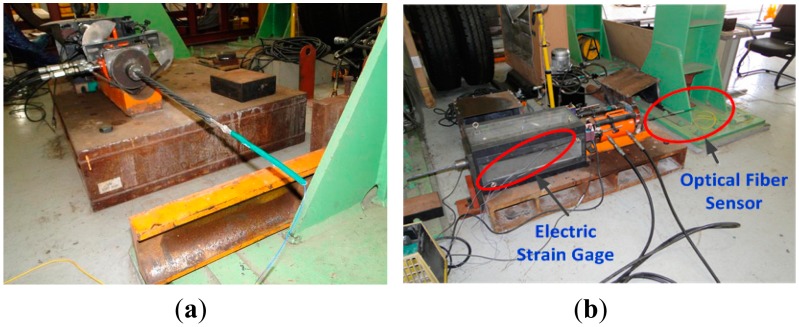
(**a**) Sensing strand specimen and (**b**) setting of strand performance test.

In order to evaluate precisely the section in which the strand develops its performance by itself, preloading of about 100 kN is applied within the elastic range of the strand to minimize the additional displacement generated in the wedge and anchorage. Figure 5 shows the sensing strand specimen and the test setup.

## 3. Results

### 3.1. Core Wire Specimens

**Figure 6.**
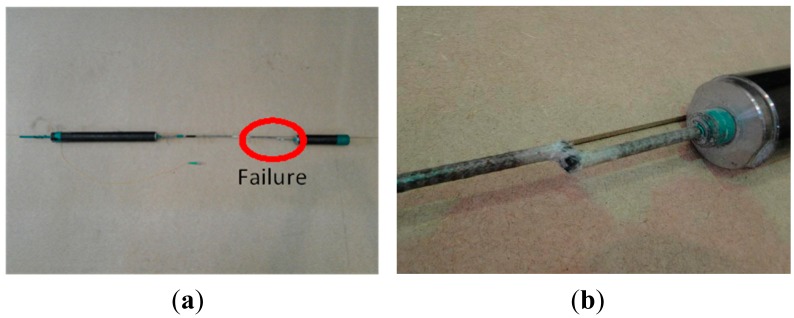
(**a**) Failure pattern of carbon core wire specimens and (**b**) detailed view.

Figure 6 shows the final failure patterns of the core wire specimens. Unlike the steel core wire, the carbon core wire behaves linearly until its brittle failure, which is the typical failure mode of carbon fiber.

**Figure 7.**
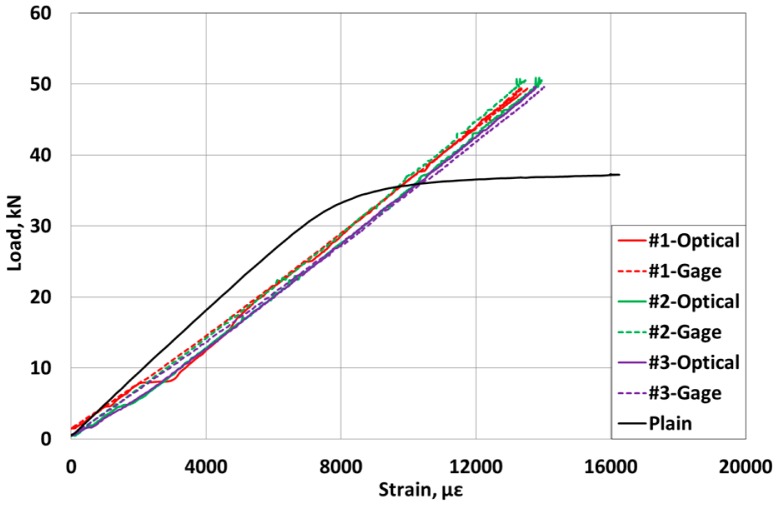
Load-strain curves of the core wire specimens of the sensing-strand.

Figure 7 plots the load-strain curves of the carbon core wire. The elastic modulus of the carbon core wire is seen to reach approximately 163 GPa, which indicates a smaller stiffness corresponding to 81.5% of that of the traditional steel core wire, but the final failure strength is about 130% of that of the steel core wire.

Besides, the strain measured by the optic sensor ranges up to 13,000–14,000 με at failure. In addition, the values of the strain measured by the optic sensor inside the specimens and the electrical resistance strain gauges at the surface of the specimens are seen to be practically identical.

### 3.2. Strand Specimens

**Figure 8.**
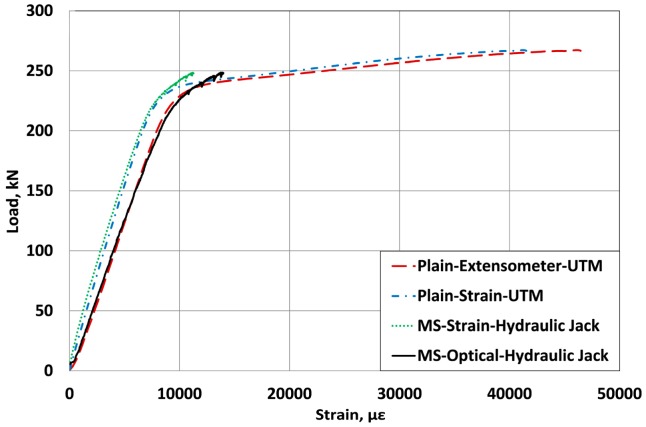
Load-strain curves of the sensing-strand (optical sensor and extensometer).

**Figure 9.**
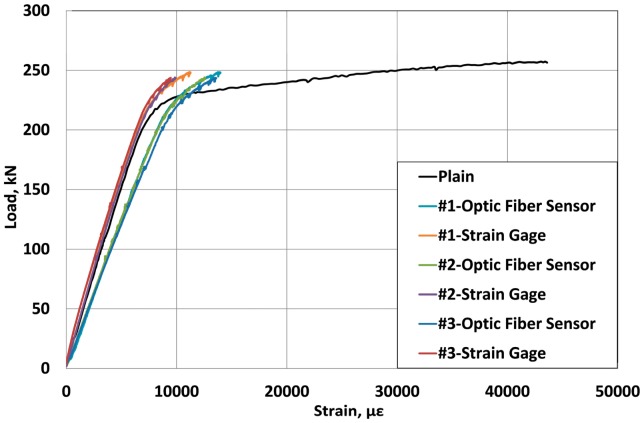
Load-strain curves of the sensing-strand (optical sensor & electrical strain gauge).

Since the tensile strength of the carbon core wire inserted in the strand is significantly superior to that of the steel core wire, even if its stiffness is lower, the outer helical wires yield first under the application of the prestress force. The yielding of the outer wires provokes a major loss of the load bearing capacity that must be then supported by the core wire, which results in the failure of the carbon core wire followed immediately by the breakage of the outer wires. Figures 8 and 9 compare the load-strain curves of the sensing-strand measured by the optical sensor, the extensometer and the electrical resistance strain gauges.

## 4. Discussion

Since the sensing-strand developed in this study is planned to replace a traditional PC strand, it must possess sufficient structural performance apart from its sensing performance. As shown in the results of Figure 8, the strand presents quasi-elastic behavior until the design prestress force even if the stiffness of the internal carbon core wire is lower than the traditional steel core wire owing to the compensation provided by the outer steel helical wires. The variation in the stiffness and strain patterns is practically identical to that of the traditional steel strand. Accordingly, the sensing-strand can be assumed to display sufficient structural performance to be applied in PSC structures.

When the strand is used to introduce prestress in the concrete structure in a real site, the value of the strain corresponding to the design prestress force is arithmetically estimated as approximately 7500 με. In Figures 7, 8 and 9, it was observed that the strain measurement range of the sensing-strand developed in this study runs around 13,000∼14,000 με, which indicates that the sensing-strand can provide measurement data over a range significantly higher than that required of the traditional strand. Accordingly, it can be concluded that the sensing-strand displays sufficient sensing performance to replace the traditional strand in real PSC structures.

**Figure 10.**
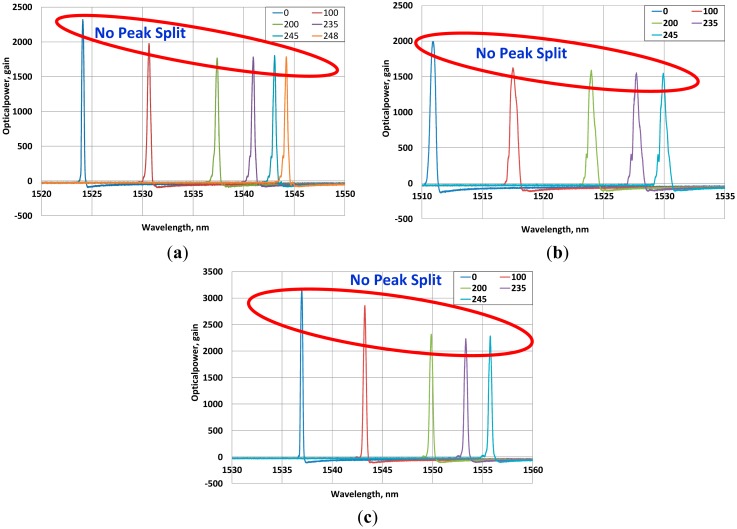
Load-spectrum change in (**a**) 20-mm; (**b**) 30-mm and (**c**) 50-mm specimens.

However, the measuring range of the sensing-strand is far larger than the well-known range of 10,000 με of the optical sensor [13]. This stresses the necessity to conduct the reliability analysis of the measurement data, which is verified by analyzing the spectrum produced by the optical sensor during loading. In order to prepare for the variation of the spectrum and difference in the sensing performance according to the length along which the optical cable and the carbon fiber are monolithically composed, specimens are fabricated with lengths of 20 mm, 30 mm and 50 mm of the monolithic section and, subjected to identical testing method. The spectrum is measured per loading stage and the analysis results are arranged in Figure 10, where the *X*-axis is the wavelength of the optical fiber and the *Y*-axis is the optical power.

The analysis of the spectrum of the optical sensor reveals the absence of a peak split of the wavelength regardless of the size of the load in each specimen. Even if sidebands occur on both sides of some spectra, their sizes remain very small. Moreover, the half-bandwidth appears to remain relatively constant and indicates a good preservation of the spectral configuration. Consequently, the data measured by the sensing-strand are verified to provide sufficient reliability.

Besides, the measured data which range far beyond the well-known measurement range of the optical fiber can be expected to extend to a higher level owing to the monolithic combination of the glass fiber of the optical sensor with the carbon fiber that exhibits significantly superior strength and stiffness that the glass fiber.

**Figure 11.**
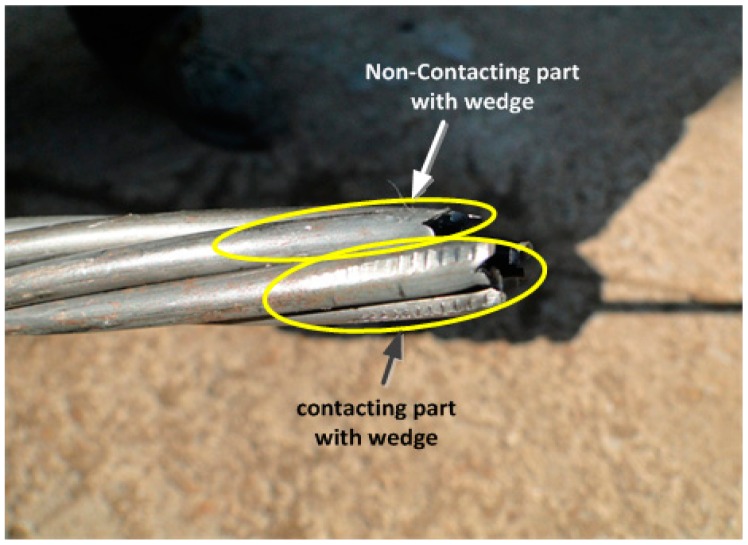
Strand-wedge contact.

Table 2 lists the strains developed when the prestress force reaches its design value of 200 kN. The strains measured by the electrical resistance strain gauges bonded on the outer helical wires show a standard deviation of 85.3 to 326.3 με per location. In addition, the strains of the specimens also exhibit large differences ranging between 6130.7 and 6897.0 με. Such differences can be explained by the differences in the fastening force of the wedge according to the surface of contact between the outer wires and the wedge as shown in Figure 11, and the difference in the orientation and status of the gauges on the outer wires.

Moreover, the stress difference computed by multiplying the difference between the maximum and minimum strains by the elastic modulus ranges between 4.2 and 15.3 MPa. The comparison of these results with the design prestress force of 200 kN reveals an error greater than 7% in the estimation of the prestress force. This confirms the inappropriateness of the method of measuring the prestress force of the strand through the outer wires and around the outer wires due to the risk of damage and the unavoidable introduction of a certain level of error. On the other hand, the optical sensor produces small differences in the strain between the specimens at 200 kN. Consequently, the measurement of the strain at the center of the core wire is recommended.

As shown throughout these results, the sensing-strand developed in this study exhibits consistent structural performance that allow it to replace the traditional PC strand, constitutes the most adequate method to measure the axial strain of the strand, and secures the reliability of the measured data. Accordingly, it can be assumed that the sensing-strength bears sufficient applicability for use in real PSC structures.

## 5. Conclusions

This study developed an innovative PC strand enabling short and long term monitoring by embedding a FBG sensor in the strand in order to effectively evaluate the prestress force of the PC strand. The tensile tests of the core wire and strand verified that this innovative strand presents structural performance equivalent or superior to the traditional PC strand. The measuring performance of the sensing-strand was also verified to be capable of measuring strain up to a range of 13,000∼14,000 με, clearly superior to the strain of 7500 με corresponding to the design prestress force of a conventional steel strand. The analysis of the spectrum conducted to examine the reliability of the measured data revealed the absence of peak splitting in each loading stage. Even if sidebands occurred around the spectrum, their size remained extremely small. The measured data appeared to provide sufficient reliability since the spectrum half-bandwidth was well maintained with larger loading. Consequently, the sensing strand developed in this study can be assumed to exhibit good applicability for real PSC structures.

## Figures and Tables

**Table 1.** Specifications of optical fiber sensor.

**Sensor**	**Specifications**	**Photograph**
FBG sensor	Strain range: 10,000 με Fusionable Wavelength: 1,510–1,590 nm	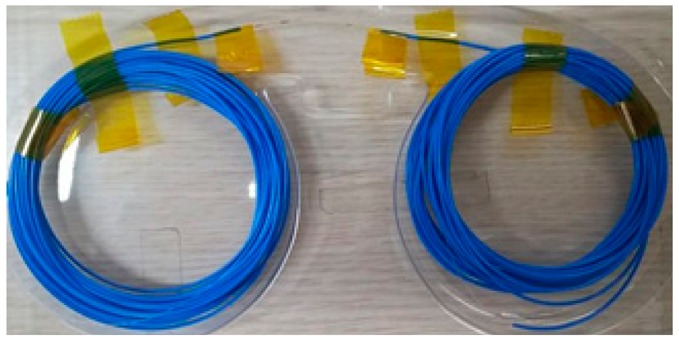

**Table 2.** Comparison of strain at prestress force of 200 kN.

**Specimen**	**Strain by Electrical Resistance Gauges on Outer Wires (με)**			**Standard Deviation**	**Stress Difference = ε_s_(max − min) × *E*_s_, MPa**	**Strain by Optical Sensor (ε_o_)**

	**0°**	**120°**	**240°**			
Basic	6969.3	6877.2	6765.5	83.3	4.7	-
#1	6287.5	6366.4	6646.2	153.9	7.2	8200.8
#2	6130.7	6711.0	6897.0	326.3	15.3	8295.5
#3	6343.9	6463.1	6552.3	85.3	4.2	8099.3
